# Anesthetic management of cesarean delivery in parturients with ruptured sinus of Valsalva aneurysm: CARE-compliant 2 case reports and literature review

**DOI:** 10.1097/MD.0000000000006833

**Published:** 2017-05-12

**Authors:** Gang Fang, Man Li, Jian Li, Li Lin, Wei Mei

**Affiliations:** a Department of Anesthesiology; b Department of Cardiology, Tongji Hospital, Tongji Medical College, Huazhong University of Science and Technology, Wuhan; c Department of Anesthesiology, Shenzhen Second People's Hospital, Shenzhen, China.

**Keywords:** case report, cesarean delivery, parturients, sinus of valsalva aneurysm

## Abstract

**Rationale:**

Ruptured sinus of Valsalva aneurysm is rare and dangerous in parturients. Few cases of ruptured SVA in pregnancy are reported, and the anesthetic management for cesarean delivery has scarcely been described.

**Patient concerns:**

A parturient at 37-week gestation complained of a sore throat and cough that started 3 days before admission, followed 1 day later by fever, dizziness, breathlessness, and palpitation on exertion. Case two at 36-week gestation complained of a 1-day history of bloating in the lower abdomen.

**Diagnoses:**

Full term and preterm parturients with ruptured sinus of Valsalva aneurysm.

**Interventions:**

Cesarean deliveries were performed with incremental epidural anesthesia technique under invasive monitoring. Surgical correction of the ruptured sinus of Valsalva aneurysms and ventricular septal defect were performed uneventfully 13 days and 7 days postpartum, respectively, for the 2 cases.

**Outcomes:**

No complications were observed in the intra- or postoperative period for both mothers and babies.

**Lessons:**

We reviewed the pertinent literature and reached the following conclusions: use of a multidisciplinary team to guide anesthetic management is helpful and necessary; and both general anesthesia and incremental epidural anesthesia can be safely used in parturients with ruptured sinus of Valsalva aneurysm.

## 1 Introduction

Cardiac disease is a major cause of maternal mortality, and about 25% of maternal cardiac deaths in the past 30 years have been due to congenital heart diseases.^[1]^ First described by Hope in 1839, sinus of Valsalva aneurysm (SVA) is a rare heart defect usually of a congenital nature, characterized by a dilatation of the coronary sinus caused by the lack of continuity between aortic media and annulus fibrosus of the aortic valve.^[2]^ Although SVA is rare in the Western world, it occurs much more commonly in male patients of Asian origin and comprises ∼0.1% to 3.5% of all congenital cardiac anomalies.^[3]^ Patients are often asymptomatic before the rupture of SVA, which is a life-threatening event and requires immediate recognition and intervention.^[4,5]^ Few cases of ruptured SVA in pregnancy are reported, and the anesthetic management for cesarean delivery has scarcely been described.^[6]^

We present our successful anesthetic management of cesarean delivery of 2 recent cases. The reporting of these cases was approved by the Institutional Review Board and both patients gave written informed consent for the report.

## 2 Methods

To identify studies for inclusion in this review, 2 authors independently searched PubMed for relevant studies published up to May 2016. The search was limited to studies conducted in humans. Only cases in English language were included. Search terms used included [“ruptured sinus of Valsalva aneurysm” OR “sinus of Valsalva aneurysm”] and [“pregnant” OR “delivery” OR “parturient” OR “cesarean”].

### 2.1 Case description

#### 2.1.1 Case 1

A 27-year-old primigravida (weight 50 kg, height 158 cm) was referred to our hospital on August 2010 with a 37-week gestation due to shortness of breath after a 3-day history of sore throat. She was asymptomatic before 36 weeks’ gestation. The patient complained of a sore throat and cough that started 3 days before admission, followed 1 day later by fever, dizziness, breathlessness, and palpitation on exertion. She reported no medical history except for a fully-recovered acute nephritis 15 years ago. On physical examination, the patient appeared acutely ill, and vital signs were temperature 37.8°C, heart rate 94 beats/min, respiratory rate 28 breathes/min, blood pressure 107/70 mm Hg, and oxygen saturation by pulse oximetry 98% on room air. Auscultation revealed a loud P_2_ and a grade 4/6 systolic murmur over the precordial region, best heard in the pulmonary area. Breath sounds were low in both lungs, without rales or rhonchi. Her functional status of New York Heart Association (NYHA) was class II–III.

Laboratory tests on the admission day were normal. Transthoracic color Doppler echocardiography revealed an 8 mm × 9 mm aneurysm in the right coronary sinus of Valsalva, protruding into the right ventricular outflow tract (RVOT), with a 4 mm-wide perforation. A high pressure left-to-right jet started from a laceration of aneurysm, the peak flow velocity (PFV) is 5.8 m/s and pressure gradient (PG) is 136 mm Hg. A mild tricuspid regurgitation was present. A 3 mm intracristal ventricular septal defect (VSD) was also present on echocardiography. The left atrium (37 mm) and left ventricle (55 mm) were enlarged. Of note, the size of right atrium (45 mm) and right ventricular (51 mm) were also enlarged. In addition, there was evidence of mild pulmonary hypertension, with an estimated pulmonary artery systolic pressure of 42 mm Hg and an estimated left ventricular ejection fraction of 69% at rest. The functional capacity of patient could not be determined due to bed rest requirement.

The patient asked for cesarean delivery without maternal or fetal indication. Four days after admission, the patient received cesarean delivery under epidural anesthesia.

#### 2.1.2 Case 2

A 22-year-old woman (G_2_P_0_, weight 57 kg, height 163 cm) with a 36-week gestation was admitted for a 1-day history of bloating in the lower abdomen on August 2010. She was diagnosed with VSD in the childhood but remained asymptomatic without treatment. There was no other significant past medical history. On physical examination, the patient showed no acute distress. Vital signs were temperature 36.5°C, heart rate 105 beats/min, respiratory rate 20 breathes/min, blood pressure 145/57 mm Hg, and oxygen saturation by pulse oximetry 98% on room air. Auscultation revealed a grade 5/6 continuous murmur along the lower left sternal border with thrill. Breath sounds were normal in both lungs. Her functional capacity was NYHA II.

Laboratory tests were normal. Transthoracic color Doppler echocardiography showed a 9 mm × 10 mm aneurysm in the right coronary sinus of Valsalva, with a 7 mm rupture into the RVOT; a 4 mm supracristal VSD with left-to-right flow at rest, mild aortic, mitral, and tricuspid regurgitation, a 4 mm pericardial effusion, enlarged left atrium (42 mm) and left ventricle (58 mm) and an estimated left ventricular ejection fraction of 63% at rest.

Because ultrasonography revealed a complete placenta previa, an elective cesarean delivery under epidural anesthesia was planned.

Both patients were managed by a multidisciplinary team of obstetricians, cardiologists, and anesthesiologists, and the cesarean deliveries were planned in a cardiac operating room with cardiopulmonary bypass capabilities on standby. Intravenous infusion of metoclopramide 10 mg and ranitidine 40 mg was used as for aspiration prophylaxis 30 minutes before the operations. Antibiotic prophylaxis of surgical infection with intravenous penicillin was started 30 minutes prior to skin incision.

Upon arrival in the operating room, both patients were monitored with a 2-channel 5-lead electrocardiography, pulse oximetry, and noninvasive blood pressure. Uterine displacement was applied by a 15° left-tilt of the operation table, and a radial arterial line and a central venous catheter via the right internal jugular vein were placed under the guidance of ultrasonography. Epidural catheter was inserted via the L_1_–L_2_ intervertebral space on the left lateral decubitus position. After a 3 mL epidural test dose (2% lidocaine without epinephrine), incremental doses of 3 to 5 mL of 2% plain lidocaine were administered every 5 minutes, to avoid systemic hypotension. A total of 19 mL and 18 mL of 2% lidocaine was used for the 2 patients, respectively, including the test dose. An upper sensory level of T_6_ was achieved, tested by sharp pinprick. A prophylactic low-dose phenylephrine infusion was part of a standard protocol for preventing hemodynamic instability during epidural anesthesia.^[7,8]^ The surgeries proceeded uneventfully without pain or discomfort, with delivery of newborns with satisfactory Apgar scores (Table 1).

**Table 1:**
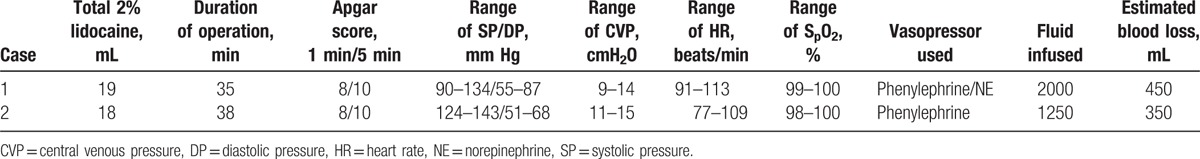
Intraoperative outcomes of our patients.

For case 1, the invasive blood pressure (IBP) abruptly decreased to 90/55 mm Hg following slow intravenous bolus injection of 5-unit oxytocin for prophylaxis against uterine atony, and marked bradycardia developed after a higher dose of phenylephrine infusion. So norepinephrine infusion was started instead immediately, at a rate of 0.05 to 0.08 μg kg^−1^ min^−1^. Intraoperative fluid therapy was guided by IBP, HR, and central venous pressure (CVP). Patient-controlled epidural analgesia of 0.2% ropivacaine was started with a background dose of 6 mL/h and 4 mL as a bolus with a 20-minute lockout interval, which was removed 48 hours later, achieving a 0 to 30 visual analog scale of pain for both patients.

After the operation, both patients were doing well without any complications, and surgical closure of VSD and correction of ruptured SVA under general anesthesia with cardiopulmonary bypass was planned. The procedures proceeded uneventfully 13 days and 7 days postpartum, respectively, for the 2 cases. Postoperatively, no residual shunt or aortic regurgitation was noted by transthoracic echocardiography at regular follow-ups. Both mothers and babies were doing well 12 months later, without any complications.

## 3 Discussion

We present 2 Chinese parturients with ruptured SVA. Both of them gave birth to a healthy baby under cesarean delivery with well-managed epidural anesthesia. Both patients received repaired surgery and they were alive with complete remission on a 1-year follow-up.

The underlying histopathological etiology of SVA is usually thought to be an inherited deficiency of connective tissue and muscular tissue in the aortic sinus. However, it can also be acquired from infection, trauma, surgery, inflammatory diseases or degenerative diseases, which usually affects more than 1 sinus of Valsalva. About 65% to 85% of SVA originate from the right sinus of Valsalva, and those originating from the noncoronary (10% to 30%) or left sinuses (<5%) are relatively rare.^[9]^ SVA frequently co-exist with other cardiac abnormalities, including VSD in 30% to 60% cases and aortic regurgitation in 44% to 50% cases, which may present a diagnostic conundrum because the diverse clinical presentations.^[4,10]^ Sakakibara and Konno^[11]^ proposed the first formal classification system for SVA in 1962. Then, Xin-jin and Xuan proposed a modified classification system in 2013.^[12]^ This modified classification system described 5 types of aneurysms. Type I protrusion and rupture into right ventricle beneath pulmonary valve; type II penetration and rupture into or just beneath crista supraventricularis of right ventricle; type III penetration and rupture into right heart adjacent to or at tricuspid annulus; type IV protrusion and rupture into right atrium; type V other rare conditions (e.g., rupture into left atrium, pulmonary artery, left ventricle, or other structures).

Clinical manifestation of an unruptured SVA may range from an asymptomatic murmur to sudden death as a consequence of right ventricular outflow obstruction, infective endocarditis, arrhythmias, myocardial infarction, and ischemia due to distortion of the coronary ostia or compression of the coronary trunk. Progressive dilatation of the SVA can lead to aortic regurgitation secondary to displacement of valve leaflets by the aneurysm.^[13]^ Associated symptoms may include dyspnea, chest pain, syncope, or congestive heart failure.^[14]^ Usually, color Doppler echocardiography is a simple and accurate noninvasive way of diagnosing ruptured SVA,^[15]^ and it was valuable in the correct diagnosis of our patients in spite of the VSD which complicated the hemodynamic change. Coronary angiography, computed tomography, magnetic resonance imaging is also valuable in the diagnosis of SVA. However, potential radiation risks must be considered.

Acute symptomatic ruptures can proceed spontaneously or are precipitated by exertion, trauma, endocarditis, or cardiac catheterization.^[9]^ Chest pain and dyspnea may precede heart failure if rupture is abrupt, but the size and the location of the shunt, rapidity of onset, and the chamber into which the rupture occurs are the major determinants of the exact hemodynamic consequences and prognosis.^[13]^ In a Texas Heart Institute study, most SVAs ruptured into the right ventricle (60%) or the right atrium (29%), only a few ruptured into left atrium (6%), the left ventricle (4%), or the pericardium (1%).^[16]^ Right-heart overload, right ventricular failure, and significant tricuspid regurgitation are frequent sequela of rupture into the right-sided chambers. Rupture of SVA into the left heart may induce acute left heart failure, which may be clinically indistinguishable from acute aortic insufficiency.^[13]^ An extracardiac rupture of the aneurysm into the pericardium might lead to a fatal cardiac tamponade.^[14]^

During pregnancy, the cardiovascular system is progressively stressed due to increased blood volume, increased heart rate, and stroke volume, and pregnancy is extraordinarily risky for women with underlying cardiac diseases.^[17]^ Histopathologic findings in human aortic media associated with pregnancy includes fragmentation of the reticulum fibers, diminished amount of acid mucopolysaccharides, and loss of the corrugation of elastic fibers in the vessel. These changes begin early in gestation and are most pronounced in the third trimester and the peripartum period.^[18,19]^ In our patients, perhaps the rupture of SVA was precipitated by the hyperdynamic state of late pregnancy coupled with hormonally induced changes in the mechanical properties of connective tissue.

Previously reported cases were identified by searches conducted on PubMed (1960 to May 2016) using the following key words: “ruptured sinus of Valsalva aneurysm” or “sinus of Valsalva aneurysm” and “pregnant” or “delivery” or “parturient” or “cesarean” or “pregnancy.” Only cases in English language were included. Twelve articles published between 1960 and 2016 were retrieved, and peripartum management of 13 deliveries in 12 patients was described. Time of publication, diagnosis and severity of ruptured SVA, mode of delivery, peripartum care, time of corrective surgery, and maternal and fetal outcome are shown in Table 2, with the clinical details of some cases not given.

**Table 2:**
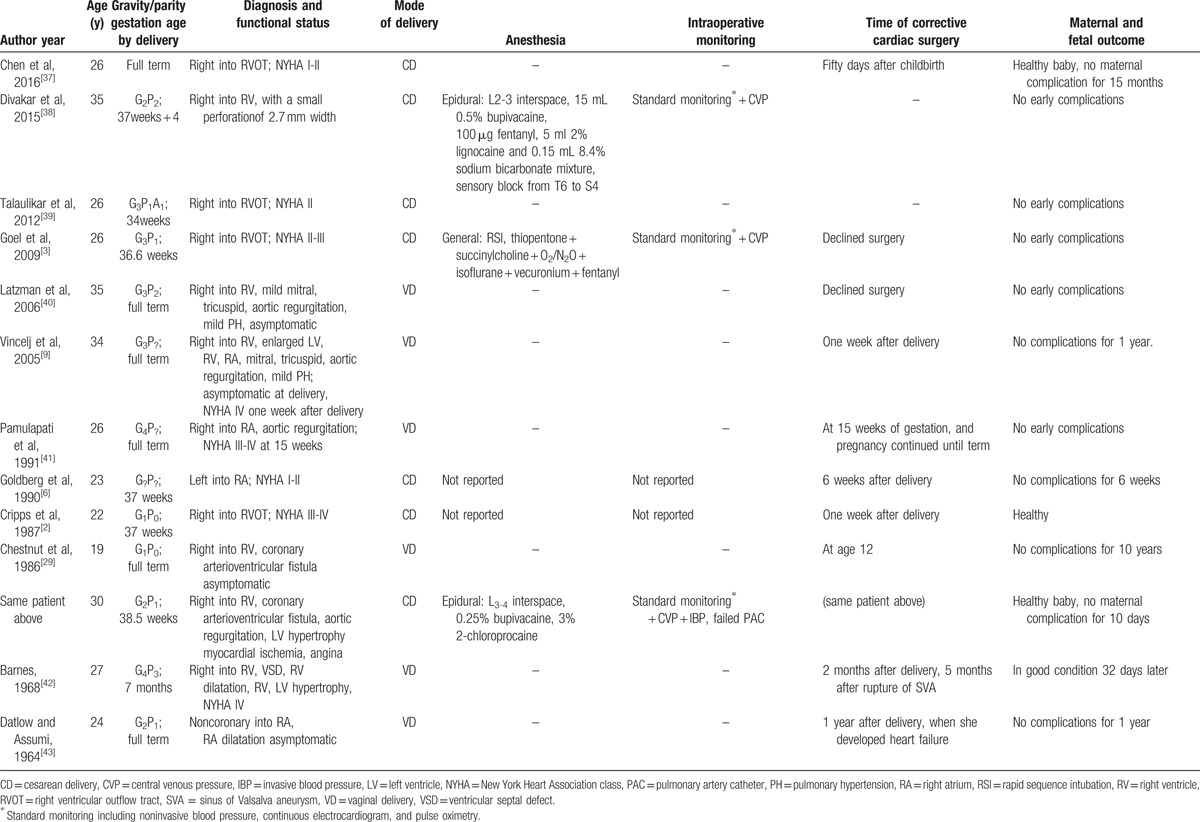
Literature review of peripartum management of patients with ruptured SVA.

Multiple studies have shown that vaginal delivery is well tolerated in most patients with cardiac disease.^[20,21]^ Cesarean delivery is usually performed for obstetrical indications only.^[22]^ However, if the patient was already hemodynamically compromised, cesarean delivery was the preferred method.^[2]^ A prospective study demonstrated that pregnant women with cardiac disease may be safe when received cesarean delivery under regional anesthesia.^[7]^ And successful outcomes have been reported with regional anesthesia for every type of cardiac pathology.^[7,23]^ For the high-risk parturients, various successful regional anesthetic management options have been reported: combined spinal epidural,^[23,24]^ spinal catheter insertion,^[25]^ or incremental epidural anesthesia.^[26,27]^ Epidural anesthesia with incremental doses is the most often advocated as the best regional technique. There is now an increasing number of case reports highlighting the use of incremental epidural anesthesia with good outcome,^[26–28]^ including in patients with ruptured SVA.^[29]^

Well-managed epidural anesthesia has the advantage of avoiding myocardial depression associated with volatile anesthetics and the stress of endotracheal intubation in general anesthesia, and causes minimal hemodynamic and respiratory disturbance. The mildly decreased SVR due to sympathetic blockade can be expected to favor forward blood flow in patients with aortic insufficiency.^[2]^ Also, the reduced SVR can alleviate the left-to-right shunting and consequent overload of the right ventricle and pulmonary vasculature due to the ruptured SVA and VSD in our patients. Incremental small boluses of 2% lidocaine without epinephrine were used to produce a T_6_ block without substantial circulatory changes. For patients with ruptured SVA, the level of neuraxial blockade involves an equilibrium between safety and comfort. Traditional T_4_ level blockade can cause bradycardia due to sympathetic blockade of cardio-accelerator fibers, which is dangerous for these high-risk patients. We aim to control the sensory blockade level to T_6_ and the procedure proceeded without significant pain or discomfort.^[30]^ Even though, the circulation side-effects of epidural anesthesia may still occur, which can be managed with carefully selected vasopressors. Phenylephrine is the first vasopressor of choice during routine cesarean delivery, and for people with cardiac disease. It is highly effective, easily titratable, and safe for the baby.^[31]^ Norepinephrine has been shown to have similar efficacy for maintaining blood pressure, with smaller incidence of bradycardia.^[32]^

In our hospital, general anesthesia is used when neuraxial anesthesia is contraindicated or in cases of life-threatening emergency with inadequate time for neuraxial anesthesia. The choice of general versus epidural-spinal anesthesia must be made after considering the patient's unique pathophysiology and in consultation with cardiologists, obstetricians, obstetric anesthesiologists, and cardiac anesthesiologists.

In these 2 cases, regurgitation of multiple valves and pericardial effusion were present, and we think vigilant monitoring of the hemodynamic status, heart sounds, and electrocardiography is essential for these patients. Continuous arterial and CVP monitoring were valuable in guiding our fluid therapy and infusion of vasopressors. Pulmonary artery catheterization is a helpful technique for intensive bedside hemodynamic monitoring, which has been believed to be necessary in patients with cardiogenic shock, for the differential diagnosis of pulmonary arterial hypertension, and for diagnosis and treatment of uncommon causes and complications of heart failure. However, its routine use is not necessary and may be associated with increased complication.^[33]^ The usefulness of a pulmonary artery catheterization in patients with ruptured SVA is not clear. It has been reported that ventricular tachycardia occurred repeatedly during attempted pulmonary artery catheterization due to a sinus of Valsalva aneurysm protruding into the right ventricle and obstructing advancement of the pulmonary artery catheter.^[34]^ Transesophageal echocardiography can provide information about intracardiac shunting and valve function, but it may not be tolerated in conscious patients. The use of transthoracic echocardiography is usually more effective and meaningful.

For patients with uncorrected ruptured SVA, the average survival time after diagnosis is 3.9 years, supporting the indication for surgical repair.^[35]^ It is not clear when corrective surgery should be performed in a pregnant woman with ruptured SVA, which involves the safety of both the mother and the fetus. Both our patients were close to full-term, so decision of cesarean delivery before ruptured SVA repair was appropriate. Had the patients presented earlier in pregnancy, the decision to terminate pregnancy would involve the balance between the risk of early delivery to the fetus and the hemodynamic consequences with continuing gestation to the mother.^[2]^ In situations in which the baby is viable, it may be in the best interest of the mother and baby for the obstetrician to perform a cesarean delivery before the cardiac surgery, with the parents’ informed consent.^[36]^ Percutaneous transcatheter closure is an effective minimally invasive alternative for ruptured SVA correction,^[5]^ and may be a good alternative for patients of earlier pregnancy. However, it was unavailable at the time in our hospital.

For our patients, close observation and early mobilization without anticoagulants for several days after delivery was allowed before surgical correction of the aneurysms, to minimize the risk of hemorrhage from the placental site. To date, there is no consensus on the timing of subsequent surgical correction of SVA. On the one hand, at very short interval, there is the risk of more complicated perioperative course and outcome of the second surgery (fading SIRS, infection, compromise of immune system, etc.), whereas on the other hand, there is always a risk of worsening of the underlying disease in the postpartum period.

## 4 Conclusion

Use of a multidisciplinary team to guide anesthetic management is helpful and necessary, and both general anesthesia and incremental epidural anesthesia can be safely used for parturients with ruptured sinus of Valsalva aneurysm.
